# Impact of Q141K on the Transport of Epidermal Growth Factor Receptor Tyrosine Kinase Inhibitors by ABCG2

**DOI:** 10.3390/cells8070763

**Published:** 2019-07-23

**Authors:** Yutaka Inoue, Takashi Morita, Mari Onozuka, Ken-ichi Saito, Kazumi Sano, Kazuhiko Hanada, Masami Kondo, Yoichi Nakamura, Tohru Kishino, Hiroshi Nakagawa, Yoji Ikegami

**Affiliations:** 1 Department of Phrmacometrics and Pharmacokinetics, Meiji Pharmaceutical University, 2-522-1 Noshio Kiyose-shi, Tokyo 204-8588, Japan; 2 Department of Pharmacy Services, Saitama Medical Center, Saitama Medical University, 1981 Kamoda, Kawagoe-shi, Saitama 350-8550, Japan; 3 Department of Medical Oncology, Division of Thoracic Oncology, Tochigi Cancer Center, 4-9-13 Yohnan Utsunomiya-shi, Tochigi 320-0834, Japan; 4 Department of Pharmacy Services, Saitama Medical University Hospital, 38 Morohongo, Moroyama-machi, Iruma-gun, Saitama 350-0495, Japan; 5 Department of Applied Biological Chemistry, Graduate School of Bioscience and Biotechnology, Chubu University, 1200 Matsumoto-cho, Kasugai-shi, Aichi 487-8501, Japan; 6 Pharmaceutical Education and Research Center Dept. of Clinical Information Evaluation Meiji Pharmaceutical University, 2-522-1 Noshio Kiyose-shi, Tokyo 204-8588, Japan

**Keywords:** ABCG2, ATP-binding cassette transporters, multidrug resistance, single nucleotide polymorphism, tyrosine kinase inhibitor

## Abstract

The ATP-binding cassette transporter ABCG2 is expressed in various organs, such as the small intestine, liver, and kidney, and influences the pharmacokinetics of drugs that are its substrates. ABCG2 is also expressed by cancer cells and mediates resistance to anticancer agents by promoting the efflux of these drugs. In the present study, we investigated the interactions between epidermal growth factor receptor tyrosine kinase inhibitors and ABCG2 by MTT assay, intracellular drug accumulation assay, and FACS. This study showed that four epidermal growth factor receptor tyrosine kinase inhibitors (EGFR TKIs) (gefitinib, erlotinib, lapatinib, and afatinib) were transported from tumor cells as substrates of ABCG2. Q141K is a common single-nucleotide polymorphism of ABCG2 in Asians. We demonstrated that the extracellular efflux of gefitinib, erlotinib, and lapatinib was reduced by Q141K, whereas afatinib transport was not affected. In addition, all four EGFR TKIs inhibited the transport of other substrates by both wild-type and variant ABCG2 at 0.1 μM concentrations. Accordingly, epidermal growth factor receptor tyrosine kinase inhibitors may induce interactions with other drugs that are substrates of ABCG2, and single-nucleotide polymorphisms of ABCG2 may influence both the pharmacokinetics and efficacy of these anticancer agents.

## 1. Introduction

ABCG2 is a membrane-associated glycoprotein that is a member of the ATP-binding cassette (ABC) transporter superfamily. ABCG2 is a half transporter and forms homodimers to actively excrete substrates from cells. It has a broad substrate spectrum and can transport various anticancer drugs, including SN-38, methotrexate, topotecan, and imatinib, which means that overexpression of ABCG2 by cancer cells causes multidrug resistance [1,2,3,4,5]. ABCG2 is not only expressed by cancer cells, but also by small intestinal epithelial cells, renal proximal tubule epithelial cells, liver cells, and capillary endothelial cells in the brain, and it influences the pharmacokinetics of drugs that are its substrates [6,7].

In recent years, there have been many reports of interactions between ABCG2 and tyrosine kinase inhibitors [1,8,9]. Gefitinib and erlotinib are epidermal growth factor receptor tyrosine kinase inhibitors (EGFR TKIs) that are substrates of ABCG2 [10,11]. A total of four EGFR TKIs (gefitinib, erlotinib, lapatinib, and afatinib) are currently in clinical use, with gefitinib, erlotinib, and afatinib being employed to treat lung cancer, while lapatinib (which inhibits both EGFR and Her2) is used for breast cancer. Afatinib is a second-generation EGFR TKI that causes irreversible inhibition of EGFR tyrosine kinase. Expression of ABCG2 has been reported in both lung cancer and breast cancer, suggesting that it may influence tumor resistance and the pharmacokinetics of EGFR TKIs [12,13,14]. Moreover, EGFR TKIs have been reported to inhibit the transport of other substrates by ABCG2, suggesting that drug interactions may occur in patients taking these anticancer agents. Recently, we found that gefitinib inhibits both ABCB1 and ABCG2, and that the antitumor activity, tumor tissue, and blood concentrations of SN-38 (the active metabolite of irinotecan) are increased in tumor-bearing mice by administration of gefitinib in combination with irinotecan [9].

More than 80 single-nucleotide polymorphisms (SNPs) of *ABCG2* have been found to date. These SNPs are thought to cause differences in the pharmacokinetics and efficacy of substrate drugs among patients since ABCG2 acts as a transporter of various drugs [15,16]. The most extensively studied SNP is Q141K (in which lysine is substituted for glutamine at position 141), which is frequently observed in Japanese and Chinese individuals [17,18]. Q141K is a germline mutation that reduces ABCG2 protein expression and impairs its transport activity in the plasma membrane [19]. It has been reported that Q141K increases the incidence of diarrhea in patients with non-small cell lung cancer receiving gefitinib therapy [20]. Thus, it seems that this SNP may modulate the effects of substrate anticancer agents, but its influence on the transport of EGFR TKIs is not well understood.

Accordingly, we performed an in vitro investigation of the interactions between EGFR TKIs (gefitinib, erlotinib, lapatinib, and afatinib) and ABCG2. We found that the Q141K variant was associated with reduced transport of gefitinib, erlotinib, and lapatinib compared with wild-type ABCG2, while it had no influence on afatinib transport. These findings suggest that Q141K may influence the pharmacokinetics of gefitinib, erlotinib, and lapatinib in patients receiving anticancer therapy.

## 2. Materials and Methods

### 2.1. Cell Lines

We used a wild-type ABCG2 (ABCG2 WT) transgenic cell line (Flp-In-293/ABCG2 WT), a Q141K transgenic cell line (Flp-In-293/ABCG2 Q141K), and a cell line in which only the vector was transferred (Flp-In-293/mock). HEK293 Flp-In cells (Flp-In-293) were transfected with the ABCG2 (WT or Q141K)-pcDNA5/FRT vector, the Flp recombinase expressing plasmid pOG44 using LipofectAmineTM-2000 (Invitrogen, Waltham, MA, USA), as described previously [19,21]. The transfected cells were selected by hygromycin B (Invitrogen) [19,21]. Flp-In-293/mock cells were prepared by transfecting Flp-In-293 cells with empty pcDNA5/FRT and pOG44 vectors in the same manner as described above [19,21]. All cells were cultured in DMEM (Wako, Osaka, Japan) containing 10% (v/v) FBS and 100 μg/mL hygromycin B at 37 °C under 5% CO_2_. Viable cell counts were determined with a hemocytometer after trypan blue staining.

### 2.2. Preparation of Cell Lysates

After culture, cells were washed with PBS and then treated with lysis buffer A (50 mM Tris-HCl (pH 7.4), 1 mM DTT, 1% (v/v) Triton X-100, and a general protease inhibitor cocktail (Nacalai Tesque, Inc., Kyoto, Japan)). Then, the samples were homogenized by being drawn up through a 27-gauge needle 10 times. After centrifugation at 800× *g* for 10 min at 4 °C, the supernatant was collected (cell lysate). The protein level of the lysate was measured using a Protein Assay Bicinchoninate Kit (Nacalai Tesque, Inc.), and then the lysate was mixed with Sample Buffer Solution with Reducing Reagent for SDS-PAGE (Nacalai Tesque, Inc.).

### 2.3. Immunoblotting Analysis

Before performing sodium dodecyl sulfate polyacrylamide gel electrophoresis (SDS-PAGE), the samples were treated with a reducing agent. After electrophoretic separation on 7.5% polyacrylamide gel, proteins were transferred to a nitrocellulose membrane by electroblotting. The membrane was incubated in skim milk overnight at 4 °C.

The following antibodies were used. The primary antibody for ABCG2 was BXP-21 (Kamiya Biomedical Company, Seattle, WA, USA; 1:2500 dilution), while the primary antibody for β-actin was C4 (Santa Cruz Biotechnology, Santa Cruz, CA, USA; 1:5,000 dilution). The secondary antibody was an anti-mouse IgG horseradish peroxidase (HRP)-linked antibody (Cell Signaling Technology, Inc., Beverly, MA, USA; 1:3000 dilution) for BXP-21 and an HRP-labeled anti-mouse IgG (H + L) antibody (Vector Laboratories, Burlingame, CA, USA; 1:10,000 dilution) for β-actin. Luminescence of HRP was developed by using Immobilon Western Chemiluminescent HRP Reagent (Millipore, Billerica, MA, USA), and then was detected with a Lumino Imaging Analyzer ImageQuant 400 (GE Healthcare, Tokyo, Japan).

### 2.4. MTT Assay

Flp-In-293/ABCG2 WT cells, Flp-In-293/ABCG2 Q141K cells, and Flp-In-293/mock cells were distributed in 96-well plates at 3000 cells/well and were cultured at 37 °C for 24 h. Then, one of the test compounds (SN-38, topotecan, cisplatin, novobiocin, or an EGFR TKI) was added to the wells and culture was continued for 72 h at 37 °C. After that, 20 µL of 2 mg/mL MTT (Nacalai Tesque, Inc.) were added to each well and incubation was continued for 4 h at 37 °C. Centrifugation was performed at 450× *g* for 10 min at 4 °C, after which the supernatant was removed, and the MTT formazan product was dissolved by adding 200 µL of DMSO to each well. Then, the absorbance was determined at 570 nm (reference wavelength: 655 nm) by using a BIO-RAD Model 550 Microplate Reader (Bio-Rad Laboratories, Hercules, CA, USA).

### 2.5. Imaging Cytometry

Flp-In-293/ABCG2 WT cells, Flp-In-293/ABCG2 Q141K cells, and Flp-In-293/mock cells were seeded in 96-well plate at 3.0 × 10^4^ cells/well and were cultured at 37 °C for 24 h. Hoechst 33342 (a substrate of ABCG2) was added to the suspension at 5 µg/mL either alone or in combination with each EGFR TKI or novobiocin (as positive control), followed by incubation at 37 °C for 18 h. Then, the intensity of Hoechst 33342 fluorescence was determined using an In Cell Analyzer 2200 (GE Healthcare; Little Chalfont, UK). Ex/Em was at 390/511 nm. A total of 30,000 events were counted for each sample.

### 2.6. HPLC

Flp-In-293/ABCG2 WT cells, Flp-In-293/ABCG2 Q141K cells, and Flp-In-293/mock cells were harvested by trypsinization and suspended in DMEM at 1.0 × 10^6^ cells/mL. Then, an EGFR TKI was added to the suspension at 20 µM, and the cells were incubated in a water bath at 37 °C or 4 °C for 0, 1, 5, 10, 30, or 60 min. After incubation, the cells were washed twice with cold PBS, 0.01 N NaOH was added, and sonication was performed to prepare lysates.

The concentration of each EGFR TKI in the lysates was measured by HPLC using an LC-2000Plus system with a PU-2089 Plus pump, CO-2067Plus column oven, UV-2075Plus UV detector, and InertSustain C18 column (5 μm 4.6Φ × 150 mm; GL Sciences, Inc., Tokyo, Japan). The mobile phase was a mixture of 0.1 M TEA-H_3_PO_4_ (pH 8): AcCN: THF at 55:45:2 (v/v/v) for gefitinib, erlotinib, and afatinib, while a 45:55:2 (v/v/v) mixture was used for lapatinib. The flow rate was 1.0 mL/min and the temperature was 30 °C. Detection of gefitinib, erlotinib, and afatinib was performed at 338 nm, while lapatinib was detected at 260 nm.

### 2.7. Statistical Analysis

Statistical analyses were performed by using Microsoft Excel 2007 software (Microsoft Co., Redmond, WA, USA). The statistical significance of differences was determined by Student’s *t*-test or one-way ANOVA. A value of *p* < 0.05 and 0.01 was considered statistically significant.

## 3. Results

### 3.1. Expression of ABCG2 and Resistance of Flp-In-293 Cells

**Figure 1:**
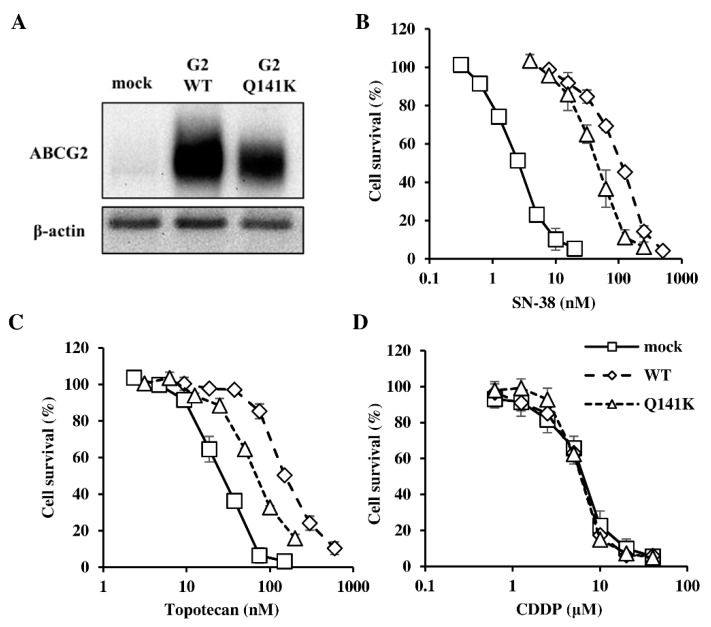
ABCG2 protein expression and transport activity in Flp-In-293 cells. (**A**) ABCG2 protein was detected by immunoblotting in Flp-In-293/mock cells, Flp-In-293/ABCG2 WT cells, and Flp-In-293/ABCG2 Q141K cells. (**B**) The cytotoxicity of SN-38, (**C**) topotecan, and (**D**) CDDP for Flp-In-293/ABCG2 WT cells, Flp-In-293/ABCG2 Q141K cells, and Flp-In-293/mock cells was examined by the MTT assay, and transport of ABCG2 substrates was also evaluated. Each drug was added at various concentrations and the cell viability was evaluated by the MTT assay after incubation for 72 h. Data are expressed as the mean ± SD. Each experiment was repeated three times.

We evaluated the expression of ABCG2 protein by immunoblotting and assessed ABCG2 transport activity by MTT assay to investigate the cellular expression and function of this protein. Expression of ABCG2 protein was weaker in Flp-In-293/ABCG2 Q141K cells compared with Flp-In-293/ABCG2 WT cells (Figure 1A). In addition, SN-38 and topotecan showed stronger cytotoxicity for Flp-In-293/ABCG2 Q141K cells than for Flp-In-293/ABCG2 WT cells (Figure 1B,C). It has been reported that cisplatin (CDDP) is not a substrate of ABCG2, and no significant differences in the cytotoxicity of cisplatin were observed among the cell lines (Figure 1D) [22]. These findings suggest that ABCG2 transport activity was reduced in Flp-In-293/ABCG2 Q141K cells compared with Flp-In-293/ABCG2 WT cells.

### 3.2. Effect of Q141K on Transport of EGFR TKIs

**Figure 2:**
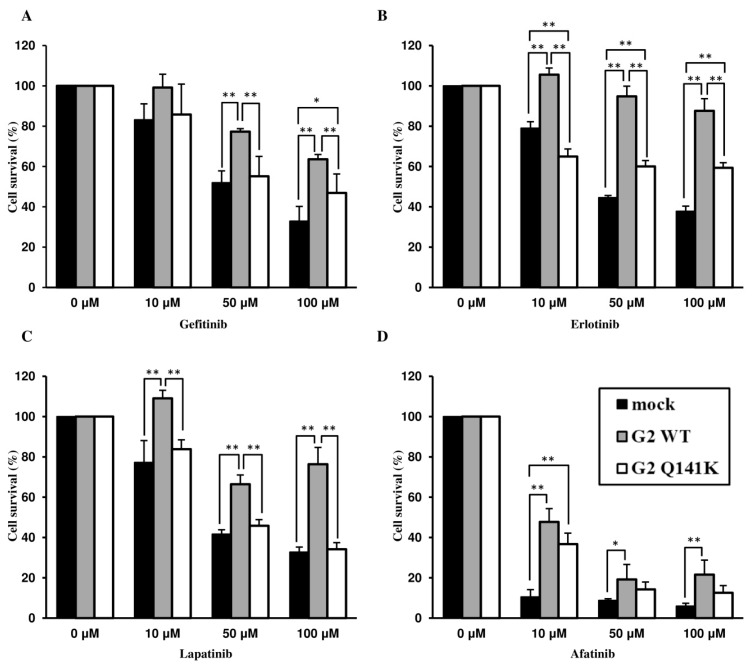
Transport of epidermal growth factor receptor tyrosine kinase inhibitors (EGFR TKIs) by ABCG2. The viability of Flp-In-293/mock cells, Flp-In-293/ABCG2 WT cells, and Flp-In-293/ABCG2 Q141K cells was evaluated by the MTT assay after incubation with EGFR TKIs: (**A**) gefitinib, (**B**) erlotinib, (**C**) lapatinib, or (**D**) afatinib. Each drug (10, 50, or 100 µM) was added to cultured cells and the MTT assay was performed after incubation for 72 h. *P*-values were determined by ANOVA and Tukey’s test (* *P* < 0.05, ** *P* < 0.01). Data are expressed as the mean ± SD. Each experiment was repeated three times.

**Figure 3:**
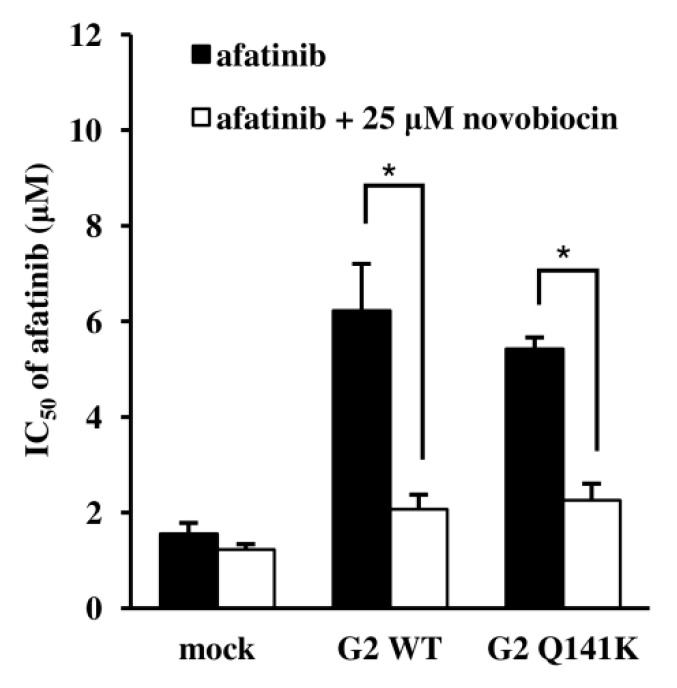
Transport of afatinib by ABCG2. The viability of Flp-In-293/mock cells, Flp-In-293/ABCG2 WT cells, and Flp-In-293/ABCG2 Q141K cells was evaluated by the MTT assay after incubation with afatinib alone or afatinib combined with 25 µM novobiocin for 72 h. *P*-values were determined by Student’s *t* test (* *P* < 0.01). Data are expressed as the mean ± SD. Each experiment was repeated three times.

**Figure 4:**
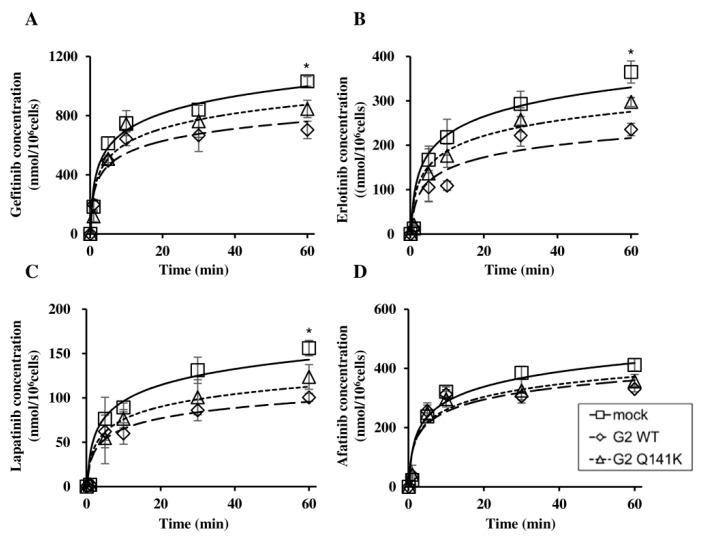
Intracellular accumulation of EGFR TKIs. Each EGFR TKI (gefitinib (**A**), erlotinib (**B**), lapatinib (**C**), or afatinib (**D**) at 20 µM) was added to Flp-In-293/mock cells, Flp-In-293/ABCG2 WT cells, and Flp-In-293/ABCG2 Q141K cells, followed by incubation for 0, 1, 5, 10, 30, or 60 min and measurement of the intracellular EGFR TKI concentration. *P*-values were determined by Student’s *t*-test (* *P* < 0.05 compared G2 WT with G2 Q141K at 60 min). Data are expressed as the mean ± SD. Each experiment was repeated three times.

After incubating Flp-In-293/mock cells, Flp-In-293/ABCG2 WT cells, and Flp-In-293/ABCG2 Q141K cells with the EGFR TKIs (gefitinib, erlotinib, lapatinib, or afatinib), we evaluated cytotoxicity by the MTT assay to examine the effect of the Q141K SNP on the transport of these anticancer agents by ABCG2. After incubation with gefitinib, erlotinib, or lapatinib, Flp-In-293/ABCG2 Q141K cells showed lower viability compared with Flp-In-293/ABCG2 WT cells (Figure 2A‒C). Incubation with afatinib reduced the viability of both Flp-In-293/ABCG2 WT cells and Flp-In-293/ABCG2 Q141K cells in a concentration-dependent manner (Figure 2D). The IC50 of afatinib was 1.56 ± 0.23 µM for Flp-In-293/mock cells, 6.23 ± 0.98 µM for Flp-In-293/ABCG2 WT cells, and 5.43 ± 0.24 µM for Flp-In-293/ABCG2 Q141K cells. When cells were incubated with afatinib in combination with an ABCG2 inhibitor (novobiocin at 25 µM), the IC50 values were 1.23 ± 0.12 µM, 2.07 ± 0.31 µM, and 3.23 ± 0.35 µM, respectively (Figure 3). Subsequently, we evaluated the intracellular accumulation of EGFR TKIs and found that the intracellular concentrations of gefitinib, erlotinib, and lapatinib were significantly higher in Flp-In-293/ABCG2 Q141K cells than in Flp-In-293/ABCG2 WT cells at 60 min (Figure 4A‒C), whereas afatinib concentrations were similar between these cells (Figure 4D). These data on intracellular drug accumulation supported the MTT assay results and suggested that Q141K affected the transport of gefitinib, erlotinib, and lapatinib but not afatinib.

### 3.3. Effects of EGFR TKIs on ABCG2 Transport Activity

**Figure 5:**
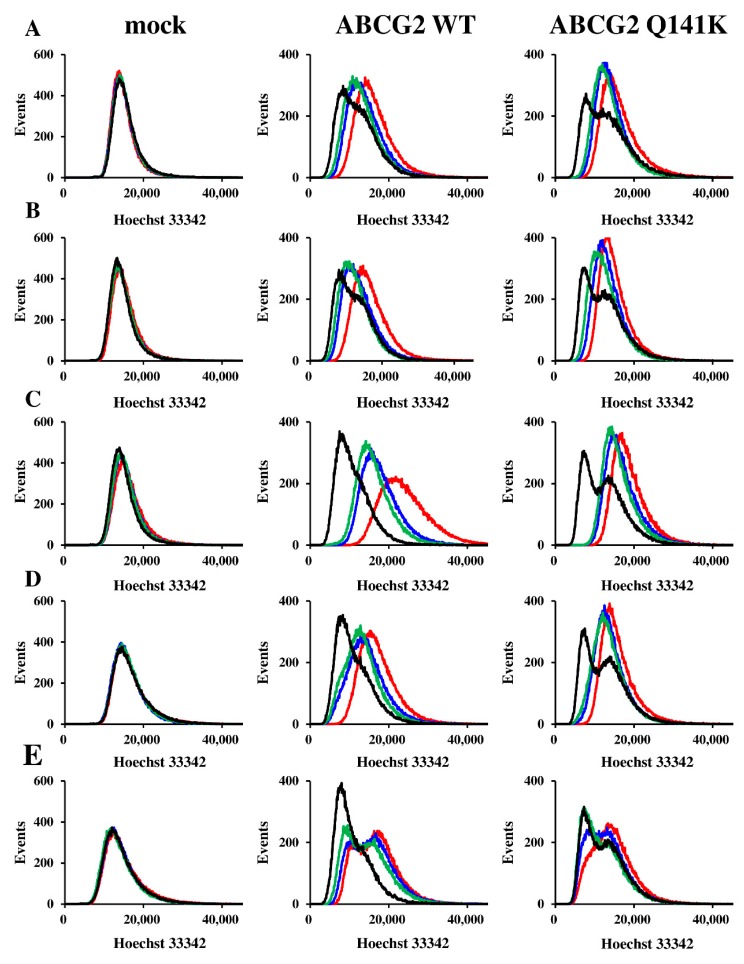
Effects of the EGFR TKIs on intracellular accumulation of Hoechst 33342. Hoechst 33342 alone or Hoechst 33342 combined with (**A**) gefitinib, (**B**) erlotinib, (**C**) lapatinib, (**D**) afatinib, or (**E**) Novobiocin was added to Flp-In-293/mock cells, Flp-In-293/ABCG2 WT cells, and Flp-In-293/ABCG2 Q141K cells. The black line indicates cells incubated with Hoechst 33342 alone. (**A**–**D**) The red, blue, and green lines indicate incubation of cells with Hoechst 33342 combined with EGFR TKIs at 5 µM, 1 µM, and 0.5 µM, respectively. (**E**) The red, blue, and green lines indicate 10 µM, 5 µM, and 1 µM novobiocin, respectively. Each experiment was repeated three times.

We investigated the intracellular accumulation of Hoechst 33342 by imaging cytometry to evaluate the effects of EGFR TKIs on ABCG2 transport activity. After incubation with Hoechst 33342 (a substrate of ABCG2), the fluorescence intensity of each cell line was determined by imaging cytometry. All four EGFR TKIs (gefitinib, erlotinib, lapatinib, and afatinib) significantly increased the mean fluorescence intensity of Hoechst 33342 in Flp-In-293/ABCG2 WT cells and Flp-In-293/ABCG2 Q141K cells (Figure 5), indicating that all of these agents inhibited ABCG2, competitively or non-competitively.

**Figure 6:**
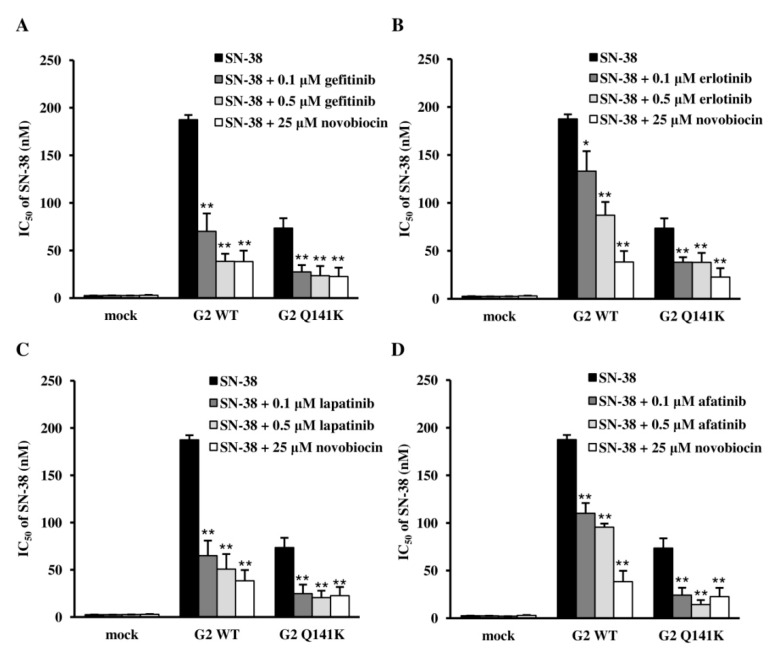
Effects of EGFR TKIs on ABCG2 transport activity. SN-38 alone or SN-38 combined with gefitinib (**A**), erlotinib (**B**), lapatinib (**C**), or afatinib (**D**) was added to Flp-In-293/mock cells, Flp-In-293/ABCG2 WT cells, and Flp-In-293/ABCG2 Q141K cells. Each EGFR TKI was added at two concentrations (0.1 µM and 0.5 µM). After incubation for 72 h, the cell viability was evaluated by the MTT assay. *P*-values were determined by ANOVA and Tukey’s test (* *P* < 0.05 and ** *P* < 0.01. versus SN-38 alone). Data are expressed as the mean ± SD. Each experiment was repeated three times.

We subsequently investigated the effects of the EGFR TKIs on transport of SN-38 (a substrate of ABCG2) by using the MTT assay. The cytotoxicity of SN-38 for Flp-In-293/ABCG2 WT cells was increased by combined incubation of the cells with any of the EGFR TKIs compared with SN-38 alone (Figure 6), and this effect was stronger with gefitinib and lapatinib than with erlotinib or afatinib. Similar to the wild-type cells, the cytotoxicity of SN-38 for Flp-In-293/ABCG2 Q141K cells was increased by combined incubation with any of the EGFR TKIs. These results suggest that drug interactions may occur in patients using EGFR TKIs and other substrates of ABCG2 (irrespective of WT or Q141K).

## 4. Discussion

ABCG2 is a key transporter related to resistance to anticancer agents and is expressed by various cancers, including lung cancer and colorectal cancer [13,23]. ABCG2 transports a variety of molecules with different structures. Its major substrates among anticancer agents include SN-38 (the active metabolite of irinotecan), topotecan, methotrexate, and doxorubicin, as well as several molecular-targeting agents such as imatinib and nilotinib [3,4,24,25]. Therefore, overexpression of ABCG2 by tumors leads to multidrug resistance.

In the present study, we examined interactions between ABCG2 and four EGFR TKIs (gefitinib, erlotinib, lapatinib, and afatinib). We found that all four EGFR TKIs interacted with wild-type ABCG2. Therefore, cancers overexpressing ABCG2 would readily excrete these anticancer agents, reducing intracellular drug concentrations and possibly decreasing their antitumor activity. It has been reported that ABCG2 expression is increased in non-small cell lung cancer lines showing resistance to gefitinib, suggesting that overexpression of this transporter is one of the mechanisms underlying resistance to EGFR TKIs [10]. Next, we evaluated the effects of the four EGFR TKIs on ABCG2 transport activity and found that these agents inhibited the transport of SN-38 by wild-type ABCG2. This suggested that tumor resistance related to overexpression of ABCG2 could possibly be overcome by administering EGFR TKIs in combination with anticancer agents that are ABCG2 substrates. We could also expect increased absorption, decreased excretion, and increased brain uptake of chemotherapy agents that are ABCG2 substrates by using EGFR TKIs to inhibit ABCG2 expression in the small intestine, kidney, and blood‒brain barrier.

Subsequently, we investigated the effect of the Q141K SNP on the transport of EGFR TKIs by ABCG2. This SNP involves the substitution of glutamine for lysine at position 141, reducing both ABCG2 protein expression and transport activity in the plasma membrane [19]. Since ABCG2 plays an important role in protection against foreign substances and influences the pharmacokinetics of many drugs, reduced ABCG2 activity could have various effects. ABCG2 promotes the extracellular efflux of various carcinogens, including 2-amino-1-methyl-6-phenylimidazo [4, 5-b] pyridine, so the risk of cancer may be increased by intracellular accumulation of carcinogens if ABCG2 activity is inhibited [26,27]. ABCG2 also transports various endogenous substances, such as bile acid and uric acid, and it has been reported that the risk of gout is increased if excretion of uric acid by ABCG2 is inhibited [28,29]. Q141K is an SNP with a high allele frequency of 30% and is common in the Japanese population, but not in African-Americans [30]. It was reported that patients bearing Q141K show increased absorption and reduced excretion of drugs that are substrates of ABCG2, leading to higher blood concentrations of substrate drugs including diflomotecan, rosuvastatin, atorvastatin, and simvastatin [7,31]. For gefitinib, imatinib, and sunitinib, Q141K has also been reported to affect the pharmacokinetics and toxicity of substrate molecular-targeting agents [32,33,34]. Thus, SNPs of *ABCG2* such as Q141K may contribute to differences among individual patients in the pharmacokinetics and effectiveness of substrate drugs. This study demonstrated that the Q141K variant reduced interaction of gefitinib, erlotinib, and lapatinib compared with wild-type ABCG2, whereas SNP did not affect the interaction of afatinib. Accordingly, the pharmacokinetics and efficacy of gefitinib, erlotinib, and lapatinib may vary in patients harboring Q141K, but it would not have this effect on afatinib. Interestingly, Rudin et al. reported that there were no significant differences in the efficacy and pharmacokinetics of erlotinib between patients with Q141K and patients with wild-type ABCG2, and that two SNPs in the *ABCG2* promoter and intron 1 (-15622C/T and 1143C/T) resulting in low protein expression of ABCG2 were associated with a higher plasma concentration of erlotinib [35]. This suggests that Q141K does not necessarily alter the pharmacokinetics and effectiveness of substrate drugs in clinical, but which drugs are influenced is currently unknown. Unlike the other three EGFR TKIs (gefitinib, erlotinib, and lapatinib), afatinib causes irreversible inhibition of EGFR tyrosine kinase by covalently binding to cysteine at position 797 [36]. We found that Q141K was not associated with reduced transport of afatinib, unlike the other EGFR TKIs. Thus, further examination of third-generation EGFR TKIs that irreversibly inhibit EGFR tyrosine kinase is needed to investigate the influence of Q141K in more detail.

Moreover, we assessed the inhibition of ABCG2 activity by EGFR TKIs, revealing that Q141K may influence interactions due to the combined administration of EGFR TKIs and substrate drugs. This was the first study to investigate the interactions between Q141K and lapatinib or afatinib.

In conclusion, we found that four EGFR TKIs (gefitinib, erlotinib, lapatinib, and afatinib) interact with ABCG2. Transport of gefitinib, erlotinib, and lapatinib is reduced by Q141K compared with wild-type ABCG2, whereas transport of afatinib is similar with both wild-type and variant ABCG2. In addition, the four EGFR TKIs inhibited the transport activity of both wild-type and variant ABCG2. These findings suggest the following conclusions: (1) overexpression of ABCG2 by cancer cells may promote resistance to EGFR TKIs, (2) the Q141K variant of ABCG2 could cause differences in the pharmacokinetics of EGFR TKIs (except afatinib), and (3) EGFR TKIs may induce drug interactions by inhibiting both wild-type and variant ABCG2.
